# *In Vivo* anti-trypanosomal activity of dichloromethane and methanol crude leaf extracts of *Dovyalis abyssinica* (Salicaceae) against *Trypanosoma congolense*

**DOI:** 10.1186/s12906-015-0809-y

**Published:** 2015-08-14

**Authors:** Belay Tadesse, Getachew Terefe, Nigatu Kebede, Workineh Shibeshi

**Affiliations:** School of Medicine, College of Medicine and Health Sciences, Debre Markos University, P.O.Box 269, Debremarkos, Ethiopia; Department of Pathology and Parasitology, College of Veterinary Medicine and Agriculture, Addis Ababa University, P.O.Box 34, Debrezeit, Ethiopia; Aklilu Lemma Institute of Pathobiology, Addis Ababa University, Addis Ababa, Ethiopia; Department of Pharmacology and Clinical Pharmacy, School of Pharmacy, College of Health Sciences, Addis Ababa University, P.O. Box 9086, Addis Ababa, Ethiopia

**Keywords:** *Dovyalis abyssinica*, Anitrypanosomal activity, Mice, *Trypanosoma congolense*

## Abstract

**Background:**

African trypanosomiasis affects both humans and livestock in sub-Saharan countries including Ethiopia. Due to limitations to current chemotherapy, there is an urgent need for the development of new, safe, cheap and effective drugs. In the present study, the leaf of *Dovyalis abyssinica* was tested for its *in vivo* antitrypanosomal activity against *Trypanosoma congolense* field isolate on mice.

**Methods:**

The leaf of *D. abyssinica* was macerated using dichloromethane and methanol. The extracts at doses of 250, 200, 150 and 100 mg/kg body weight were administered intraperitonealy daily for 7 days to mice infected with *T. congolense*. Following administration, parasitemia, packed cell volume, rectal temperature, body weight and survival time were monitored.

**Results:**

Administration of dichloromethane and methanol extracts at 250 and 200 mg/kg reduced (*p* < 0.05) parasitemia and rectal temperature, and improved (*p* < 0.05) PCV, mean body weight, and mean survival time compared to dimethylsulfoxide treatment.

**Conclusion:**

Crude dichloromethane and methanol leaf extracts of *D. abyssinica* displayed anti-trypanosomal activity that may serve as lead for the development of effective alternative antitrypanosomal drugs.

## Background

The genus *Trypanosoma* causes potentially fatal human and animal trypanosomiasis in Africa and South America. The protozoal species *Trypanosoma brucei rhodesience* and *T.b. gambience* cause a fly-born severe Human African Trypanosomiasis (HAT) or sleeping sickness in Africa probably since many centuries ago [1, 2].

*T. congolense, T. vivax and T.b. brucei* cause African Animal trypanosomiasis (AAT) [3]. The occurrence of animal trypanosomiasis coincides with the distribution of tsetse fly vectors which includes the regions between latitudes 14°N and 29°S [4]. The disease is the major constraint to livestock productivity. It causes a great economic loss in the livestock industry with an estimated 3 million cattle death annually. Estimated direct production losses in cattle to African farmers are between 1 and 4 billion US dollars per year [5]. In Ethiopia, it has been described as a major impediment to the livestock development and agricultural production [6].

Despite the development of vector control tools and vaccine candidates over the years, the control of trypanosomiasis has continued to rely heavily on the use of trypanocidal drugs. However, effectiveness of current treatment is limited by many factors [7–9], necessitating the search for new improved drugs. Moreover, because those in the lower socioeconomic class are disproportionately affected by the disease, there is a great need for the development of not only less toxic and more effective drugs but also affordable agents [10, 11].

*Dovyalis abyssinica* (A. Rich) Warb. (Salicaceae) is a spiny evergreen shrub or tree containing of dovyalicin-type alkaloids, dovyalicin E and dovyalicin F, Dovyalicin A [12]. The plant displayed antibacterial and antifungal activity [13]. Recently, Nibret and Wink [14] reported on the *in vitro* anti-trypanosomal activity of the plant against *T. b. brucei*. Hence, this work was conducted to evaluate the *in vivo* anti-trypanosomal effects of crude dichloromethane and methanol leaf extracts of *D. abyssinica* on the most pathogenic East AAT*, T. congolense*.

## Methods

### Chemicals

Analytical grade solvents and reagents used in this work were; dichloromethane (DCM) (BDH laboratory supplies, England), methanol (MOH) (Sigma Aldrich, Germany), diethyl ether (Blulux Laboratories, India), diminazene aceturate (DA) (BerenilÒ, Hoechst, Germany), dimethylsulphoxide (DMSO) (BDH laboratory supplies, England).

### Preparation of plant extracts

The fresh leaves of *D. abyssinica* were collected in February, 2013, from Debrezeit, about 47.9 km Southeast of Addis Ababa, Ethiopia. Identification and authentication of the plant was done at the National Herbarium, Department of Biology, Addis Ababa University, Addis Ababa, where a voucher specimen was deposited (collection number BT001/13). The fresh plant material was rapidly washed under running tap water, shade air dried at room temperature until brittle, and grounded to coarse powder using laboratory pestle and mortar. The powdered plant material was extracted using two solvents; DCM and MOH as used by [12, 14]. To obtain DCM extracts, 200 g were macerated in 2000 ml of DCM in conical flask for three days. Similarly, to obtain MOH extract 200 g of powder were macerated in 2000 ml of MOH. The residue left after maceration was successively extracted twice with the same medium separately. For each of the two solvents extracts were collected in separate flasks and left undisturbed before being filtered through a sterile filter paper (wattman No. 1) into a clean conical flask. The filtrate was dried by evaporating the solvents using oven. The dried extracts were weighed and placed in refrigerator at 4 °C until needed.

### Experimental animals

Swiss albino mice (20–35 g) of both sexes aged 10–12 weeks were obtained from the breeding colony of school of pharmacy, Addis Ababa University. The mice were housed in polypropylene cages and maintained under room temperature and 12 h light and dark cycle. The animals were left under controlled conditions at least for one week to acclimatize them before conducting any experimental procedure. Usage of mice in experiment was in accordance with Institute for Laboratory Animals’ Resources (ILAR) Guideline [15], and was approved by research and ethics committee of the School of Pharmacy, Addis Ababa University.

### Isolation of *T. congolense*

*T. congolense* field isolate was collected from Illibabur Zone, Dabu woreda, Sebategna district, about 523 km Southwest of Addis Ababa. Cattle were screened for the presence of *T. congolense* using the buffy coat technique or Murray method [16]. Presence of *T. congolense* was identified based on motility and further confirmed by National Tsetse and Trypanosomiasis Control Centre (NTTCC) in Bedelle following thin blood smear preparation. They were fixed by MOH and stained with Giemsa stain and read using an oil immersion objective (40–50× for scanning, 100× for identification of trypanosomes). The morphology of the trypanosome in the stained field was compared with that of reference species [17]. Blood was then drawn from the jugular vein of the positive cattle using heparinized vacutainer tube and injected (0.5 ml in two divided doses) intraperitonealy to apparently healthy mice and rats and transported to Aklilu Lemma Institute of Pathobiology, Addis Ababa University. After establishment of infection, the organisms were maintained by serial passages in mice until required as described by [18].

### Acute toxicity study

Acute toxicity test was carried out according to the Organization for Economic Co-operation and Development (OECD) guidelines for Testing of Chemicals number 420 [19] and Lorke’s method [20] on female mice aged 10–12 weeks weighing 20–35 g. The test was initiated with a sighting study treating one female mouse for each of the two extracts in a stepwise procedure with the fixed doses 300 and 2000 mg/kg. Oral 300 mg/kg of both extracts did not cause any signs of toxicity in mice throughout the monitoring period. However, a single 2000 mg/kg oral dose of each extracts produced death of a mouse. Median lethal dose was estimated using twenty adult female mice grouped in to eight groups performed in two phases [20]. At 10, 100, and 1000 mg/kg IP doses there were no noticeable signs of toxicity. However administration of 1600 mg/kg dichloromethane and methanol extracts caused the death of a mouse. The LD_50_ for both plant extracts as calculated using the Lorke’s formula:

$$ LD50=\sqrt{\mathrm{aXb}} $$

(where a = least dose that killed a mouse, while b = highest dose that did not kill any mice) was 1,265 mg/kg body weight. Based on this result four doses (100, 150, 200 and 250 mg/kg) were selected for the *in vivo* studies.

### Preliminary phytochemical screening

Standard screening tests of the crude extracts were carried out for secondary metabolites according to the methods discussed in the literature [21].

#### Test for alkaloids

To 1 ml of test solution, few drops of Dragendorff’s and Mayer’s reagent were added and formation of precipitate indicates a positive result.

#### Test for saponins

About 0.5 g of the extract was dissolved in 10 ml of distilled water in a test tube. The test tube was stoppered and shaken vigorously for 30 s and allowed to stand in a vertical position and observed over 30 min. Formation of a “honey comb” froth over the surface of liquid and persistence after 30 min indicates presence of saponins.

#### Test for tannins

To 2 ml of water diluted sample 3 drops of 10 % ferric chloride were added. The formation of bluish-black color denotes the presence of tannins.

#### Test for sterols and terpenes

To 1 ml of test solution, few drops of concentrated sulphuric acid in a slant position was added and left standing for an hour. The formation of a brown ring at interphase indicates the presence of sterols and terpenes.

#### Test for flavonoids

To 1 ml of the test solution, 3mls of 10 % sodium hydroxide was added followed by 3mls of 10 % HCl. The formation of a yellow color on addition of sodium hydroxide, which disappeared on addition of the HCl, indicates the presence of flavonoids.

#### Test for steroids

The test for steroids was done by the Liebermann acid test. A portion of the extract was treated with drops of acetic anhydride. Concentrated H_2_SO_4_ was carefully added to the side of the test tube. The presence of a brown ring at the boundary of the mixture was taken as positive result.

#### Test for phenolic compounds

To 2 ml of filtered solution 1 ml of 1 % ferric chloride (FeCl_3_ and 1 ml of potassium ferrocyanate (K_4_Fe(CN)_6_) were added. Formation of bluish green color indicated the presence of phenolic compounds.

### Parasite inoculation and extract administration

A total of fifty (50) apparently healthy mice of both sexes were randomly grouped into 10 groups (DCM-250 mg/kg, DCM-200 mg/kg, DCM-150 mg/kg, DCM-100 mg/kg, MOH-250 mg/kg, MOH-200 mg/kg, MOH-150 mg/kg, MOH-100 mg/kg, DA, and DMSO) of five mice of both sexes each. Infected blood containing 2 × 10^6^ trypanosomes/ml was collected from donor mice by cardiac puncture under diethyl ether anesthesia and diluted with phosphate buffered saline (PBS) to contain 10^3^ trypanosomes/ml. All groups of mice were then infected intraperitoneally (IP) with 0.2 ml of infected blood/PBS [18]. The animals were left to develop parasitemia and 12 days post parasite challenge [22], when the parasitemia level reached 1.58 × 10^7^ trypanosomes/ml, Groups DCM-250 mg/kg, DCM-200 mg/kg, DCM-150 mg/kg, and DCM-100 mg/kg were administered with doses at 250, 200, 150 and 100 mg/kg body weight (BW), respectively, of the DCM extract, while groups MOH-250 mg/kg, MOH-200 mg/kg, MOH-150 mg/kg, and MOH-100 mg/kg received respective MOH extracts of 250, 200, 150, 100 mg/kg BW IP once daily every morning for 7 days. The extracts and diminazene aceturate were separately dissolved and reconstituted in 10 % DMSO. Whereas infected mice in group DA received 28 mg/kg of diminazene aceturate [23]. The last group was administered with DMSO to serve as a negative control.

### Determination of parasitemia

Parasitemia was monitored by examination of blood drawn from the tail of mice microscopically at ×400 magnification using the “Rapid Matching” method of Herbert and Lumsden [24]. Briefly, the method involves microscopic counting of parasites per field in pure blood. Logarithm values of these counts were obtained by matching with the table of Herbert and Lumsden [24]. Monitoring of parasitemia (average taken from two independent laboratory technicians’ data) was performed every other day until the 14^th^ day post-treatment initiation. For the assessment of anti-trypanosomal effect of the extracts, the level of parasitemia in the treated animals was compared to that of the control animals [25].

### Determination of packed cell volume

Packed Cell Volume (PCV) was determined using microhaematocrit centrifuge and microhaematocrit tube reader. Microhaematocrit centrifuge, fitted with head capable for carrying 24 capillary tubes and a revolution of 11, 000 rpm for five minutes was used. The heparinized capillary tube was ¾ filled with blood samples obtained from the tail vein of the mice. The end of the tube was sealed and excess cleared off using cotton wool. The filled tubes were placed in a slot in the centrifuge head with sealed end outward. A special scale, the microhaematocrit reader was used to obtain the PCV percentage and the reading recorded [26]. PCV was monitored on day of parasite inoculation, treatment initiation, and 7^th^ and 14^th^ post-treatment initiation.

### Determination of rectal temperature

Rectal temperature was measured by digital rectal thermometer (Mettler Toledo, Switzerland) per rectum on the day of parasite inoculation, day of treatment commencement and every other day thereafter for 2 weeks [27].

### Determination of body weight

The BW of each mouse in all groups was recorded following the period of fasting on the day of parasite challenge, day of treatment initiation, and every other day for 2 weeks by a sensitive digital weighing balance (Mettler Toledo, Switzerland) [27].

### Determination of mean survival time

Mortality was monitored daily and the number of days from the time of inoculation of the parasite up to death was recorded for each mouse in the treatment and control groups throughout the follow up period for 6 weeks [28].

### Statistical analysis

All data generated during the course of the research were expressed as mean ± standard deviation (SD) and analyzed statistically by one way ANOVA followed by Tukey’s multiple comparison tests to determine statistical significance. The level of significance for the differences between means within group was computed by student’s *t* test. Data analysis was performed using Statistical Package for Social Science (SPSS) version 16. *P* values less than 0.05 were considered statistically significant.

## Results

### Phytochemical constituents of *D. abyssinica*

Phytochemical screening tests performed on the leaf extracts of *D. abyssinica* revealed the presence of alkaloids, sapponins, phenolic compounds, flavonoids, and steroids (Table 1).

**Table 1:** Phytochemicals of crude DCM and MOH leaf extracts of *D. abyssinica*

Phytochemical constituents	DCM extract	MOH extract
Alkaloids	+	+
Sapponins	+	+
Tannins	−	−
Phenolic compounds	+	−
Flavonoids	+	+
Sterols and terpenes	−	−
Steroids	−	+

Key: + Present; − Absent

### Effect of extracts on parasitemia

Treatment with the extracts resulted in reduction in the level of parasitemia generally between days 14 and 22 compared to the negative control, in which there was a progressive rise in parasitemia throughout the 2 weeks monitoring period. Higher doses of DCM extract (250 and 200 mg/kg) significantly (*p* < 0.05) reduced the parasite load between D14 and D20, compared to the lowest doses (DCM-150 mg/kg and DCM-100 mg/kg) and the DMSO-treated control. Especially, DCM-250 mg/kg was shown to reduce parasitemia level to approximately 12 (10^1.08^) trypanosomes/ml at D20, though failed to totally clear the trypanosomes from the blood, and displayed comparable activity to diminazene aceturate (*p* > 0.05) from D18 to D20. Compared to DCM-250 mg/kg, DCM-200 mg/kg displayed higher activity which failed to reach statistical significance (*p* > 0.05), except at D16 (*p* < 0.05). Even though the activity was lower than (*p* < 0.05) the two higher doses, DCM-150 mg/kg significantly (*p* < 0.05) controlled the progression of parasitemia compared to the lowest dose (100 mg/kg) and infected DMSO-treated groups. Still DCM-100 mg/kg-treated was statistically superior (*p* < 0.05) to DMSO-treated group in reducing parasitemia (Table 2).

**Table 2:** *In vivo* anti-trypanosomal activity of dichloromethane crude leaf extract of *D. abyssinica* on parasitemia of *T. congolense* infected mice

Rx	D(mg/kg)			Parasitemia level (log number/ml)					
		D12	D14	D16	D18	D20	D22	D24	D26
DCM	250	6.96 ± .25	6.66 ± .39^bcd^	5.70 ± .37^a^	2.16 ± 3.04^bcd^	1.08 ± 2.41^bcd^	2.16 ± 2.96^bcd^	3.24 ± 2.96^a^	4.32 ± 2.41
	200	6.66 ± .58	6.48 ± .54^cd^	6.12 ± .45^cd^	4.5 ± 2.52	3.24 ± 2.98^cd^	3.24 ± 2.98^cd^	5.52 ± .16	5.88 ± .16
	150	6.66 ± .58	6.72 ± .27^cd^	6.54 ± .33^cd^	6.12 ± .40	5.88 ± .34	5.70 ± .30	5.94 ± .39	6.06 ± .39
	100	6.90 ± .47	7.14 ± .58	7.32 ± .62	7.32 ± .62	7.20 ± .60	7.86 ± .49	8.22 ± .40	8.46 ± .25
DA	28	7.08 ± .54	.00 ± .00	.00 ± .00	.00 ± .00	.00 ± .00	.00 ± .00	1.08 ± 1.0	2.16 ± 1.32
DMSO	-	7.08 ± .16	7.26 ± .25	7.48 ± .30	7.74 ± .25	7.74 ± .13	8.04 ± .25	8.04 ± .25	8.46 ± .25

Values are Mean ± SD; *p* < 0.05; *N* = 5; ^a^compared to all but diminazene aceturate; ^b^compared to DCM 150 mg/kg; ^c^compared to DCM 100 mg/kg; ^d^compared to DMSO

*Rx* Treatment, *D* Dose, *DCM* Dichloromethane extract, *DA* Diminazene aceturate, *DMSO* Dimethylsulfoxide, *D* Day, *D12* Day of treatment initiation

On the other hand, only MOH-250 mg/kg and MOH-200 mg/kg were shown to significantly reduce parasitemia level from D14 to D20 compared to the lowest two doses (150 and 100 mg/kg) and DMSO-treated groups. Within this period, compared to MOH-200 mg/kg the parasitemia reduction was shown to be higher in the MOH-250 mg/kg -treated group, though failed to reach statistical significance (*p* > 0.05) except at D20 (*p* < 0.05). MOH-250 mg/kg displayed statistically similar (*p* > 0.05) parasitemia reduction to diminazene aceturate at D18 through D22. Unfortunately, all the MOH extracts were not able to totally clear *T. congolense* from the blood of infected mice*.* Even, the lowest two doses (MOH-150 mg/kg and MOH-100 mg/kg) was statistically indifferent from DMSO (*p* > 0.05) (Table 3). However, treatment with 28 mg/kg diminazene aceturate temporarily cleared *T. congolense* from the blood within 2 days post-treatment initiation which reached statistical significance (*p* < 0.05) compared to all extracts-, except DCM-250 mg/kg and MOH-250 mg/kg, and DMSO treated groups. Relapse in this group was recorded in all mice after D24. The comparative analysis revealed that DCM extract appeared to be superior to the MOH extract in reducing parasite burden. Mice treated with DCM-250 mg/kg had continually reduced parasitemia level from D14 to D20 which was further kept on average at lowest level up to the end of monitoring period. This was statistically significant (*p* < 0.05) compared to MOH-250 mg/kg (on D16) and MOH-200 mg/kg (on D16, D18, D20, and D22). MOH-250 mg/kg was significantly superior (*p* < 0.05) in reducing parasitemia compared to DCM-150 mg/kg (D16, D20 and D22) and DCM-100 mg/kg (D16-D22). Though high parasitemia reduction was observed with DCM extract compared to similar dose of MOH extract, the difference failed to reach statistical significance (*p* > 0.05), except on D16.

**Table 3:** Anti-trypanosomal activity of crude methanol leaf extract of *D. abyssinica* on parasitemia of mice infected with *T. congolense*

Rx	D(mg/kg)			Parasitemia level (log number/ml)					
		D12	D14	D16	D18	D20	D22	D24	D26
MOH	250	7.08 ± .16	6.84 ± .25^bcd^	6.24 ± .25^bcd^	3.30 ± .13^bcd^	1.08 ± 2.55^a^	2.16 ± 2.96^a^	4.38 ± 2.45^bcd^	5.52 ± .16
	200	7.02 ± .16	6.78 ± .16^bcd^	6.48 ± .16^bcd^	5.94 ± .13	5.58 ± .16	5.76 ± .25	6.00 ± .21	6.12 ± .34
	150	7.02 ± .27	7.20 ± .3.7	7.32 ± .27	7.14 ± .25	7.26 ± .25	7.44 ± .25	7.56 ± .25	7.68 ± .34
	100	7.08 ± .16	7.32 ± .16	7.74 ± .25	7.98 ± .34	8.10 ± .37	8.28 ± .34	8.22 ± .27	8.40 ± .21
DA	28	7.08 ± .54	.00 ± .00	.00 ± .00	.00 ± .00	.00 ± .00	.00 ± .00	1.08 ± 1.0	2.16 ± 1.32
DMSO	-	7.08 ± .16	7.26 ± .25	7.48 ± .30	7.74 ± .25	7.74 ± .13	8.04 ± .25	8.04 ± .25	8.46 ± .25

Values are mean ± SD; *N* = 5; *p* < 0.05; ^a^compared to all but DA; ^b^compared to 150 mg/kg; ^c^compared to 100 mg/kg; ^d^compared to DMSO

*MOH* Methanol extract, *Rx* Treatment, *D* Dose, *DA* Diminzene aceturate, *DMSO* Dimethylsulfoxide, *D* Day, *D12* Day of treatment initiation

### Effect of extracts on packed cell volume

**Fig. 1:**
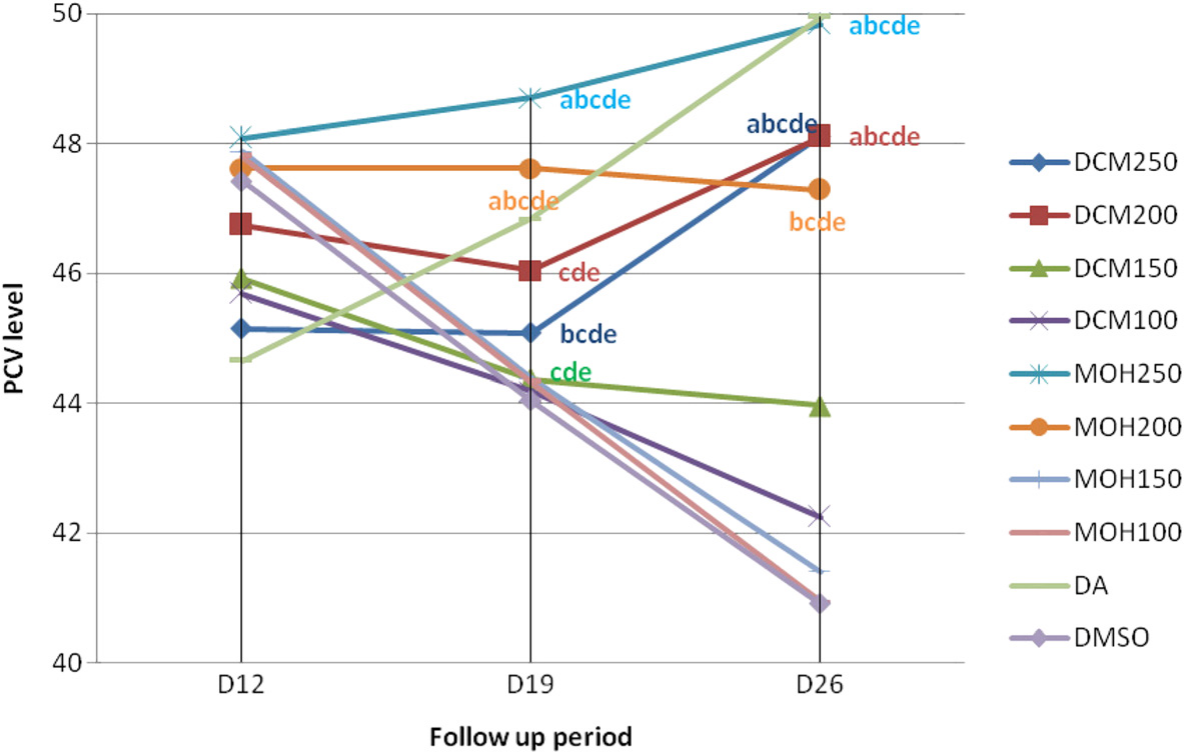
*In vivo* anti-trypanosomal activity of crude dichloromethane and methanol leaf extracts of *D. abyssinica* on packed cell volume of mice infected with *T. congolense.* Values are Means, *N* = 5; *p* < 0.01; ^*a*^compared to DCM150; ^*b*^compared to DCM100; ^*c*^compared to MOH150; ^*d*^compared to MOH100; ^*e*^compared to DMSO; *DCM* Dichloromethane extract, *MOH* Methanol extract, *DA* Diminazene aceturate, *DMSO* Dimethylsulfoxide, *PCV* Packed cell volume, *D* Day, *D12* Day of treatment initiation

Figure 1 shows that *T. congolense* caused a significant PCV reduction (*p* < 0.05) in infected mice as the average value was found to drop from 49.04 ± 0.30 to 50.50 ± 0.50 on the day of parasite inoculation to 44.64 ± 0.42 to 48.08 ± 0.14 on the day of treatment initiation. Seven days post-treatment initiation the PCV level significantly (*p* < 0.01) improved in those groups which received DCM-250 mg/kg compared to those treated with the DCM-150 mg/kg and DCM-100 mg/kg and the negative control. Similarly, compared to the negative control, DCM-200 mg/kg-treated mice had improved PCV (*p* < 0.01). However, mice treated with DCM-150 mg/kg and DCM-100 mg/kg doses had a consistently reduced PCV values, though statistically indifferent (*p* > 0.05) from those treated with DCM-200 mg/kg.

The PCV improvement brought by DCM-250 mg/kg and DCM-200 mg/kg were statistically similar (*p* > 0.05) to each other. On D22, PCV was further improved significanty (*p* < 0.01) in mice treated with DCM-250 mg/kg and DCM-200 mg/kg compared to those treated with DCM-150 mg/kg and DCM-100 mg/kg and the negative control. Likewise, 7 days post-treatent initiation MOH-250 mg/kg and MOH-200 mg/kg treated animals showed improved PCV level (*p* < 0.01) compared to those treated with MOH-150 mg/kg, MOH-100 mg/kg, and negative control whose PCV declined continuously. This effect of MOH-250 mg/kg and MOH-200 mg/kg was significantly high (*p* < 0.01) compared to MOH-150 mg/kg, MOH-100 mg/kg, and negative control during the end of the monitoring day (D26), though higher PCV increment (*p* < 0.05) was obtained from MOH-250 mg/kg than MOH-200 mg/kg. Comparative analysis revealed that comparable doses of DCM extracts were superior to MOH extracts on D19 and vice versa on D26, but the difference failed to reach statistical significance (*p* > 0.05). Despite the significant enhancement in mean PCV level at these highest doses, it was significantly lower compared to that brought by diminazene aceturate (*p* < 0.05) on D19 and D26.

### Effect of extracts on rectal temperature

**Fig. 2:**
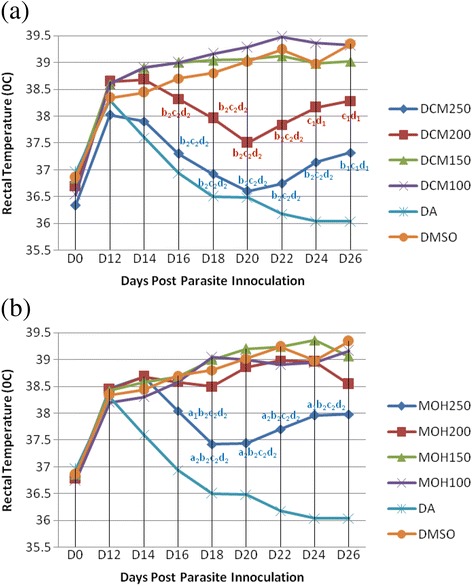
Rectal temperature change of *T. congolense* infected mice treated with crude dichloromethane (**a**) and methanol (**b**) leaf extract of *D. abyssinica*. Values are Means, *N* = 5; ^1^ *p* < 0.05; ^2^ *p* < 0.01; ^*a*^against 200 mg/kg; ^*b*^against 150 mg/kg; ^*c*^against 100 mg/kg; ^*d*^against DA; *DCM-250150 mg/kg* Dichloromethane extract 250 mg/kg, *DCM200* Dichloromethane extract 200 mg/kg, *DCM150* Dichloromethane extract 150 mg/kg, *DCM100* Dichloromethane extract 100 mg/kg, *DA* Diminazene aceturate, *DMSO* Dimethylsulfoxide, *D0* Day of parasite inoculation, *D12* day of treatment initiation

The mean rectal temperature at the day of infection (36.34 ± 0.25 to 36.96 ± 0.10) was elevated following infection (38.02 ± .24 to 38.64 ± 0.33), which indicated fever was manifested (p < 0.05) in mice infected with *T. congolense* (Fig. 2a and b)*.* Mice treated with DCM-250 mg/kg and DCM-200 mg/kg produced pronounced fever reduction (*p* < 0.05) from D16 until the end of monitoring period compared to those treated with DCM-150 mg/kg, DCM-100 mg/kg, and DMSO. Whereas the reduction in rectal temperature caused by these two doses was comparable (*p* > 0.05), DCM-250 mg/kg was superior to DCM-200 mg/kg on D16-D26. Despite this, the rectal temperature was shown to slowly rise after D22 (Fig. 2a). By contrast, MOH-200 mg/kg was unable to significantly reduce rectal temperature (*p* > 0.05) compared to DMSO. Only MOH-250 mg/kg ameliorated temperature elevation from D16 to the final monitoring day, which reached statistical significance (*p* < 0.05) compared to all doses of MOH and the negative control. However, rectal temperature started to rise on D22 (Fig. 2b). Comparative analysis indicated that DCM-250 mg/kg and DCM-200 mg/kg were superior to respective MOH-250 mg/kg and MOH-200 mg/kg doses in ameliorating rectal temperature elevation, though the difference failed to reach statistical significance (*p* > 0.05). In spite of this none, except DCM-250 mg/kg on D20, of these doses was comparable (*p* < 0.01) to 28 mg/kg diminazene aceturate in preventing rectal temperature rise associated with the infection.

### Effect of extracts on body weight

**Fig. 3:**
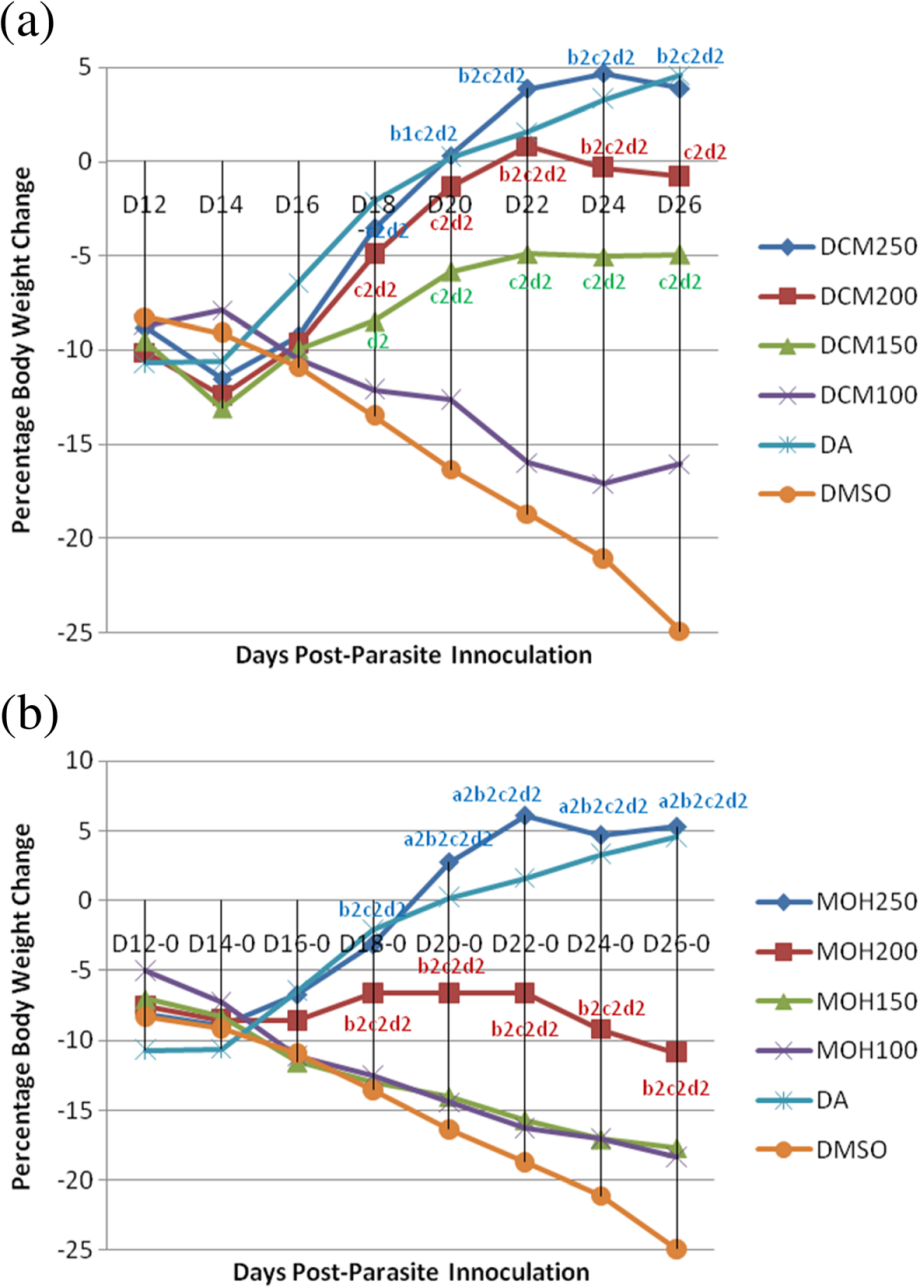
Percentage Body Weight change of *T. congolense* infected mice treated with crude dichloromethane (**a**) and methanol (**b**) leaf extracts of *D. abyssinica*. Values are Means, *N* = 5; ^1^ *p* < 0.05; ^2^ *p* < 0.01; ^*a*^against 200 mg/kg; ^*b*^against 150 mg/kg; ^*c*^against 100 mg/kg; ^*d*^against DA; *DCM250* Dichloromethane extract 250 mg/kg; *DCM200* Dichloromethane extract 200 mg/kg, *DCM150* Dichloromethane extract 150 mg/kg, *DCM100* Dichloromethane extract 100 mg/kg, *DA* Diminazene aceturate, *DMSO* Dimethylsulfoxide, *D0* Day of parasite inoculation, *D12* Day of treatment initiation

Before treatment commencement all groups show 7.5–9.15 % weight loss associated with parasitemia. However, treatment with the crude extracts prevented this loss in BW particularly at 250 and 200 mg/kg dose levels of DCM extract and 250 mg/kg of the MOH extract compared to the negative control. There were no detectable difference in BW improvement among these doses as well as between the extracts and the standard (28 mg/kg) throughout the monitoring period (Fig. 3a and b). Compared to the negative control, groups treated with all doses of DCM extract, except 100 mg/kg, significantly (*p* < 0.05) improved BW of infected mice throughout the monitoring period starting from D18. Within this follow up period BW improvement in the groups treated with DCM-250 mg/kg and DCM-200 mg/kg was not significantly different (*p* > 0.05), though better increment was produced by DCM −250 mg/kg (Fig. 3a). On the other hand, only MOH-250 mg/kg and MOH-200 mg/kg were able to significantly improve BW compared to the control group (*p* < 0.05), though MOH-250 mg/kg was statistically superior (*p* < 0.01) to MOH-200 mg/kg in improving BW of mice. By contrast, there was consistent BW lose in mice treated with MOH-150 mg/kg and MOH-100 mg/kg (Fig. 3b). Comparative analysis revealed that significant (*p* < 0.05) BW improvement was observed in mice treated with MOH-250 mg/kg (6.1 %), DCM-250 mg/kg (4.7 %), and diminazene aceturate 28 mg/kg (4.6 %) compared to those treated with MOH-200 mg/kg on days 22, 24 and 26. However, the difference in BW improvement produced by these doses were statistically insignificant (*p* > 0.05).

### Effect of extracts on mean survival time

**Fig. 4:**
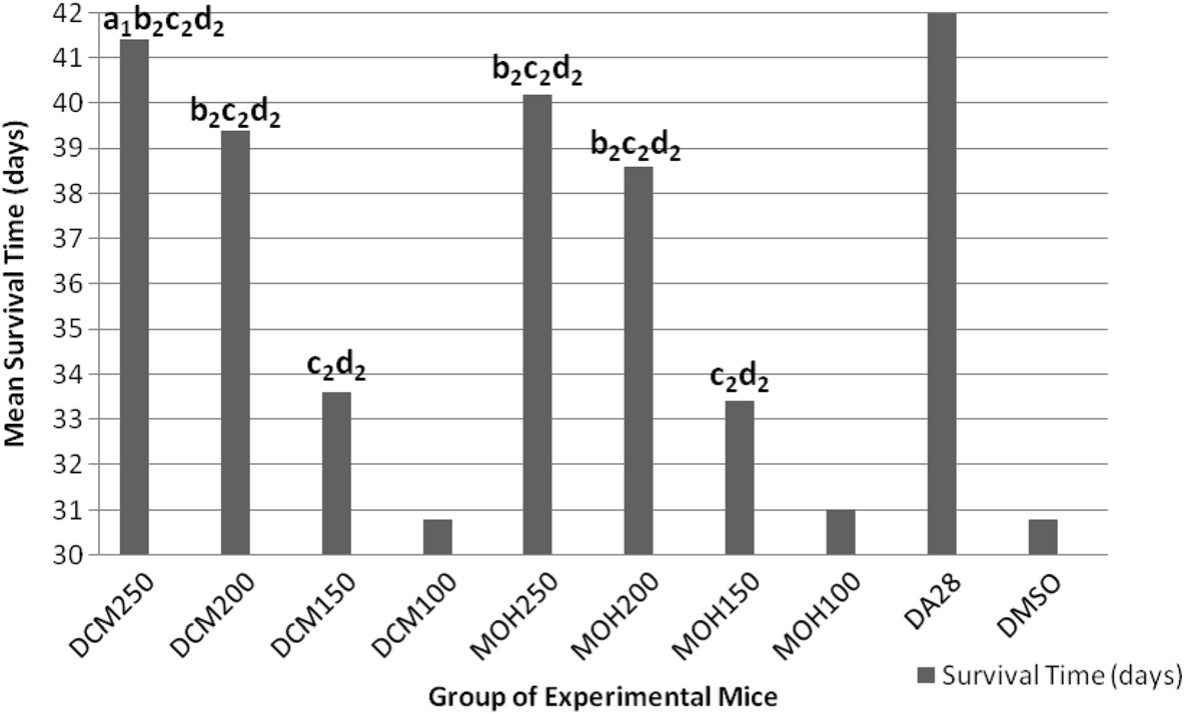
*In vivo* anti-trypanosomal activity of dichloromethane and methanol crude leaf extracts of *D. abyssinica* on mean survival time of mice infected with *T. congolense.* Values are Means, *N* = 5; ^1^ *p* < 0.05; ^2^ *p* < 0.01; ^*a*^compared to DCM200 and MOH200; ^*b*^compared to DCM150 and MOH150; ^*c*^compared to DCM100 and MOH 100; ^*d*^compared to DMSO; *DA* Diminazene aceturate, *DCM* Dichloromethane extract, *MOH* Methanol extract, *DMSO* dimethylsulfoxide

Higher doses of DCM extracts (250 and 200 mg/kg) significantly (*p* < 0.05) prolonged mean survival time of infected mice compared to that conferred by treatments with DCM-150 mg/kg, DCM-100 mg/kg, and DMSO which was below 34 days. Similarly, mice treated with MOH-250 mg/kg and MOH-200 mg/kg survived longer time than those treated with lower MOH doses (150 and 100 mg/kg) and DMSO. Significantly longer (*p* < 0.05) survival time (41.40 ± 0.24 days) was recorded in mice treated with DCM-250 mg/kg compared to those treated with MOH-250 mg/kg (40.20 ± 0.49), DCM-200 mg/kg (39.40 ± 0.24) and MOH-200 mg/kg (38.60 ± 0.51) which were statistically indifferent (*p* > 0.05) amongst themselves. All mice treated with 28 mg/kg diminazene aceturate survived until the end of the monitoring period of 42 days. Out of 40 mice treated with both extracts, only three (two DCM-250 mg/kg and one MOH-250 mg/kg treated) mice survived up to the end of the monitoring period (Fig. 4).

## Discussion

In this study the activity of crude DCM and MOH leaf extracts of *D. abyssinica* were studied in terms of *in vivo* parameters, including parasitemia, packed cell volume, rectal temperature, BW and mean survival time of infected mice.

Higher doses (250 and 200 mg/kg) of both extracts substantially reduced parasitemia level in mice, despite the failure to completely clear bloodstream form of the parasite. The result is consistent with other reports [25, 28] that medicinal plants reduce parasitemia. The effect might be attributed to the presence in them of one or more of secondary metabolites which may exert their effects through the additive or synergistic action of several chemical compounds acting at a single or multiple target sites associated with a physiological process [29, 30]. Even though detailed characterization and isolation of different compounds that could be responsible for the observed activity was not carried out, preliminary phytochemical screening indicated the presence of alkaloids, phenolic compounds, flavonoids, sapponins, and steroids. Several possible mechanisms, therefore, working separately or in concert may account for the observed effect [31].

*D. abyssinica* contains spermidine alkaloids [12], also confirmed in the present study, that may in part account for the observed anitrypanosomal activity of this plant [14]. The aryl moiety in the spermidine molecule interacts with the hydrophobic region of trypanothione reductase so that the spermidine would adopt a non-extended bound conformation [32], could be responsible for the observed effect of the alkaloid. Besides, the extracts may increase oxygen consumption and stimulation of hydrogen peroxide production in the protozoan cell [33]. Hence, the parasite might have been exposed to oxidative stress that couldn’t be dealt with the inhibited trypanothione pathway. Interestingly, spermidine in part resembles the chemical structure of pentamidine [14]. Binding to nucleic acids in DNA and RNA, or promotion of cleavage of the parasite’s circular DNA in a manner similar to that of topoisomerase II inhibitors might have also been responsible [34].

But this may not rule out the actions of the other secondary metabolites. Flavonoids targeting the replicating form of trypanosomes which are totally dependent on glycolysis for energy production [35] might have been responsible mechanisms. It may also be proposed that phenolic compounds, though only found in DCM extract, might have killed the parasite through specific inhibition of the glycerol-3-phosphate-dependent mitochondrial oxygen consumption or formation of reactive oxygen species acting as a pro-oxidant [36]. Moreover, steroids should not be neglected in this regard since sterols, such as vernoguinosterol and vernoguinoside, have been reported to have anti-trypanosomal activities [37]. On the other hand, although the anti-trypanosomal activity of saponins is controversial, saponins with detergent properties can dissolve in biomembranes and disturb their fluidity and the function of membrane proteins of parasites [38].

At similar *in vivo* doses DCM extract was superior to its MOH counterpart, which is consistent with previous report [14]. This may be credited to the presence of phenolic compounds in the DCM, but not in the MOH, extract as these compounds were shown to exhibit anti-trypanosomal activity [39]. Other report has shown similar observation that superior activity was obtained from the DCM plant extracts [28].

The observation that parasitemia was relatively elevated and that trypanosomes were persistently present in blood of extract treated animals after withdrawal of treatment (day 12, 14) may suggest recovery of the parasites from the suppressive effect of the extracts. This resurgence in mean parasitemia may be due to the waning effect of the treatment [28] or the release of trypanosomes from the tissues which can occur when treatment is delayed or the dose rate is inadequate [22]. Despite the temporary clearance of blood stream forms of trypanosomes, relapse occurred in all mice treated with diminazene aceturate at day 12 post-treatment initiation. This observation is not surprising as resistance to diminazene aceturate has been widespread in South West Ethiopia [40].

Consistent with reduction in parasitemia, it is apparent from results of the present study that administration of both extracts at higher doses (250 and 200 mg/kg) led to remarkable improvement of anemia, evident from the significant difference in the levels of the PCV of the extract treated animals and those treated with DMSO. The result is consistent with previous works [23, 28]. The increase in PCV after treatment may, therefore, be due to mechanisms associated with reduction in trypanolytic crisis which enhances red blood cells damage and destruction leading to anemia [41]. This, in turn, may be mediated by extracts ability to eliminate parasites from the blood, probably by reaching the site of action or rapid metabolization [42], or neutralization of the toxic metabolites produced by trypanosomes [43]. Certain components of the plant might have helped stabilize the membrane of erythrocytes; specifically, the anti-oxidant or free radical scavenging properties of phenolic compounds and flavonoids [44], may play vital roles in this regard.

Animals infected with trypanosomes characteristically exhibit fever shown in the present study by the occurrence of febrile peaks which reflected the response to successive waves of parasitemia that the high body temperature itself is detrimental to the trypanosomes [45]. However, following treatment with high doses (250 and 200 mg/kg) of both extracts pyrexia was reduced with attendant rise in packed cell values. The body temperature set point in the hypothalamus, which was changed under the influence of pyrogenic stimuli released during infection [46], might have, then, been kept in check.

Further deduction of the anti-trypanosomal activity of the plant can be made from the weight gain observed in mice treated with the higher (250, 200 mg/kg) doses of extracts. In the present study, infected mice manifested weight loss and similar findings have been reported in *T. congolense* infected West African dwarf goats [47] and in mice infected with *T. congolense* [28]. The weight gain might be linked to the treatment-induced reduction of parasitemia, which otherwise would lead to depressed appetite and food intake during fever peaks. Fever reduction might, therefore, have decreased synthesis of protein that would occur at the expense of muscle protein catabolism and loss in body weight [48]. The increased supply of oxygen and nutrients because of the improved PCV level may also be an important factor in the weight gain.

Compared to DMSO treatment, administration of higher doses (250 and 200 mg/kg) of both extracts conferred prolongation of lives of mice. The result agrees with other reports [49, 28] that medicinal plants prolong survival of trypanosome infected mice. This could be related to effects of the active compound found in *D. abyssinica* on red blood cells and/or antioxidant activity. The amelioration of anemia, which is primarily responsible for the death of infected animals [49], shown by the ability of these extracts to improve PCV may have then rescued the lives of mice. Additionally, the improved survival of trypanosome infected mice could be due to the effects of *D. abyssinica* leaf extracts on pathogenesis of the disease. Indeed polyphenols in tea have been shown to attenuate cytokine induced [50] and also nitric oxide inflammation [51], both of which initiate the pathological effects of trypanosomiasis [49].

## Conclusion

In the present study, administration of higher curative doses (250 and 200 mg/kg) of both extracts considerably displayed *in vivo* activity as evidenced by reduction in parasitemia level, and rectal temperature and enhanced packed cell volume, body weight, and survival time. Therefore, it could be concluded that crude dichloromethane and methanol leaf extracts of *D. abyssinica* displayed anti-trypanosomal activity that may serve as lead for the development of effective alternative antitrypanosomal drugs*.* However, bioactivity-driven isolation of *D. abyssinica* components and further antitrypanosomal activity studies are necessary to evaluate the pharmacological importance of the plant.

## Abbreviations

DCM: Dichloromethane
MOH: Methanol
DA: Diminazine aceturate
*T. congolense*: *Trypanosoma concolense*
*D. abyssinica*: *Dovyalis abyssinica*
PCV: Packed cell volume
